# Genes encoding agrin (*AGRN*) and neurotrypsin (*PRSS12*) are associated with muscle mass, strength and plasma C-terminal agrin fragment concentration

**DOI:** 10.1007/s11357-022-00721-1

**Published:** 2023-01-07

**Authors:** Jedd Pratt, Laura Whitton, Anthony Ryan, Thorhildur Juliusdottir, Jackie Dolan, Judith Conroy, Marco Narici, Giuseppe De Vito, Colin Boreham

**Affiliations:** 1 Institute for Sport and Health, University College Dublin, Dublin, Ireland; 2 Genuity Science, Dublin, Ireland; 3 Department of Biomedical Sciences, CIR-Myo Myology Centre, Neuromuscular Physiology Laboratory, University of Padova, Padua, Italy; 4 Genuity Science, Reykjavik, Iceland

**Keywords:** Sarcopenia, Genes, Neuromuscular junction, Muscle strength, Muscle mass, Agrin

## Abstract

Although physiological data suggest that neuromuscular junction (NMJ) dysfunction is a principal mechanism underpinning sarcopenia, genetic studies have implicated few genes involved in NMJ function. Accordingly, we explored whether genes encoding agrin (*AGRN*) and neurotrypsin (*PRSS12*) were associated with sarcopenia phenotypes: muscle mass, strength and plasma C-terminal agrin fragment (CAF). PhenoScanner was used to determine if *AGRN* and/or *PRSS12* variants had previously been implicated with sarcopenia phenotypes. For replication, we combined genotype from whole genome sequencing with phenotypic data from 6715 GenoFit participants aged 18–83 years. Dual energy X-ray absorptiometry assessed whole body lean mass (WBLM) and appendicular lean mass (ALM), hand dynamometry determined grip strength and ELISA measured plasma CAF in a subgroup (*n* = 260). Follow-up analyses included eQTL analyses, carrier analyses, single-variant and gene-burden tests. rs2710873 (*AGRN*) and rs71608359 (*PRSS12*) associate with muscle mass and strength phenotypes, respectively, in the UKBB (*p* = 8.9 × 10^−6^ and *p* = 8.4 × 10^−6^) and GenoFit cohort (*p* = 0.019 and *p* = 0.014). rs2710873 and rs71608359 are eQTLs for *AGRN* and *PRSS12*, respectively, in ≥ three tissues. Compared to non-carriers, carriers of rs2710873 had 4.0% higher WBLM and ALM (both *p* < 0.001), and 9.5% lower CAF concentrations (*p* < 0.001), while carriers of rs71608359 had 2.3% lower grip strength (*p* = 0.034). *AGRN* and *PRSS12* are associated with muscle strength and mass in single-variant analyses, while *PRSS12* has further associations with muscle strength in gene-burden tests. Our findings provide novel evidence of the relevance of *AGRN* and *PRSS12* to sarcopenia phenotypes and support existing physiological data illustrating the importance of the NMJ in maintaining muscle health during ageing.

**Supplementary Information:**

The online version contains supplementary material available at 10.1007/s11357-022-00721-1.

## Introduction

The progressive degradation of skeletal muscle mass and strength known as sarcopenia [1] is perhaps the most functionally significant facet of the ageing phenotype. Diagnosed as the simultaneous presence of low muscle mass and low muscle strength [2], sarcopenia imposes multiple adverse health outcomes, such as falls [3], mobility limitations [4] and a subsequent loss of independence among older adults [5]. Although considerable effort has been given to developing therapeutic and preservative strategies in recent years, sarcopenia prevalence remains high, affecting 40% of adults over the age of 80 years [6]. Further still, the burden of sarcopenia is anticipated to rise in coming decades in parallel with societal ageing. With this in mind, there is an urgent need to further elucidate the biological foundations of sarcopenia, as doing so may help facilitate an enhancement of current preventative protocols and a subsequent reduction in sarcopenia prevalence.

Considering heritability estimates of 46–76% for muscle mass [7] and 32–67% for muscle strength [8], it is clear that genetic factors are likely to serve a central role in sarcopenia pathogenesis. However, despite several recent genome-wide association studies (GWAS) [9–12], the genetic architecture and heritability of sarcopenia remain largely unknown. Although GWAS have identified potentially relevant risk loci for sarcopenia, the level of phenotypic variance explained by these variants is relatively low, with values of < 1% [10], 4.4% [11], 13% [9] and 15.5% [12] reported to date. Moreover, there is a notable lack of harmony between GWAS data and the physiological understanding of the mechanisms underpinning muscle degradation. Indeed, despite increasing physiological data suggesting that the dismantling of the neuromuscular junction (NMJ) is a principal contributor to sarcopenia [13, 14], few genes involved in NMJ health have been identified through GWAS. In order to better target treatment and prevention strategies, the pertinence of specific genes linked with the underlying aetiological mechanisms of the disease needs to be established.

Although sarcopenia is recognised as a multifaceted disease [15–19], a diminished re-innervative potential induced by NMJ dysfunction has been proposed as a primary underlying mechanism [20, 21]. During ageing, the rhythmic cycle of denervation and re-innervation that normally occurs throughout the lifespan becomes unbalanced, whereby the rate of re-innervation cannot match that of denervation. This shift results in a progressive degeneration of motor units, contributing to the dramatic loss of muscle strength and mass observed during ageing [20, 22]. In this regard, it is well accepted that the integrity of the NMJ, the communicatory link between motoneuron and muscle fibre, is crucial for maintaining the dynamic cycle of denervation and re-innervation. Accordingly, in the present study we sought to investigate the relevance of two candidate genes involved in NMJ function, to sarcopenia.

Firstly, *AGRN* located on chromosome 1p36 encodes agrin, a heparan sulphate proteoglycan that is potently involved in NMJ formation and maintenance [23]. Specifically, agrin is a pivotal mediator of the stabilisation of pre-synaptic structures and the precise aggregation of acetylcholine receptors (AChRs) on the post-synaptic membrane [23]. Furthermore, agrin acts as a potentiator of the sophisticated agrin-LRP4-MuSK signalling cascade, a crucial modulator of synaptic communication [24]. Importantly, agrin is cleaved and deactivated by the neuronal protease, neurotrypsin, subsequently releasing a 22-kDa C-terminal agrin fragment (CAF) into circulation [23]. Correspondingly, blood concentrations of CAF are indicative of NMJ dismantling, whereby higher levels indicate increased degradation. Interestingly, studies have reported elevated levels of CAF in sarcopenic populations compared to healthy controls [13, 25], supporting the importance of NMJ integrity to muscle health. Secondly, *PRSS12* located on chromosome 4q26 encodes neurotrypsin, which is responsible for the deactivation of agrin and the release of CAF into the bloodstream. With this in mind, it seems logical that *PRSS12* would be associated with circulating CAF levels and other sarcopenia-related phenotypes through its role in NMJ degradation. This hypothesis is supported by studies that demonstrate an overexpression of neurotrypsin to invoke severe NMJ fragmentation and premature sarcopenia in mice [26, 27], and another demonstrating that an injection of neurotrypsin-resistant agrin reverses many sarcopenic characteristics in mice over-expressing neurotrypsin [28].

Evidently, although physiological studies have reported promising evidence supporting the relevance of agrin and neurotrypsin to sarcopenia, few studies have investigated whether this relevance remains at the gene level. Accordingly, this study aimed to explore whether *AGRN* and/or *PRSS12* are associated with sarcopenia phenotypes (muscle mass, strength and plasma CAF levels).

## Methods

This study combines original whole genome sequence (WGS) data and detailed clinical phenotyping obtained from the GenoFit cohort, with existing UK Biobank (UKBB) data obtained from PhenoScanner and Genebass databases.

### GenoFit cohort

The GenoFit study was a large dual-site, cross-sectional analysis of individuals aged 18–92 years residing in Ireland that took place between September 2017 and October 2020 [29]. A total of 10,546 individuals participated in a once-off assessment, during which biological samples were collected and an extensive suite of health, lifestyle and fitness measurements was performed. To limit the potential of population stratification, the sample for the present study was refined to include 6715 unrelated individuals of Irish ancestry (males: *n* = 2997; females *n* = 3718) (methods used to refine sample are outlined in detail later in this section). Informed consent was obtained for all participants upon enrolment, and ethical approval was granted by University College Dublin’s (UCD) Human Research Ethics Committee.

### Phenotyping and sequencing of GenoFit cohort

A SECA (SECA, Hamburg, Germany) stadiometer and weighing scales were used to measure height and body mass, respectively, with participants dressed in light clothing and without footwear. Body mass index (BMI) was calculated as body mass divided by height (kg/m^2^). Grip strength was measured using a digital Jamar hand-held dynamometer (JLW Instruments, Chicago, IL, USA), according to a previously described protocol [29]. The dynamometer was configured so that the handle and middle phalanx formed a 90° angle. In a standing position with the arm positioned by their side, participants performed two maximal attempts (≥ 3 s) with each hand. The average of the highest score from each hand was used in the analysis. Dual energy X-ray absorptiometry (DXA) (Lunar Prodigy, GE Healthcare Technologies, USA) was used to measure whole body lean mass (WBLM) and appendicular lean mass (ALM). Appendicular lean mass was determined as the combined lean mass of the limbs. Level of physical activity and education were assessed through a self-reported questionnaire. More specifically, physical activity levels were assessed by asking: ‘How many days per week do you do at least 30 min of exercise that increases your breathing and heart rate (e.g. brisk walking, jogging, cycling, swimming)?’ Level of education was assessed by asking: ‘What is the highest level of education you have completed to date (no formal education, primary, lower secondary, higher secondary, third level or postgraduate)?’.

Blood samples were collected by experienced phlebotomists through venepuncture of the median cubital vein and vacutainers containing ethylenediaminetetraacetic acid (BD Vacutainer®). Plasma was extracted from a sub-group of 260 samples through centrifugation at 4000 rpm for 10 min at 4 °C. All samples were stored at − 80 °C until analysis. Plasma CAF concentrations were measured using a readily available enzyme-linked immunosorbent assay (ELISA) kit (#ab216945, Abcam, Cambridge, UK) according to the manufacturer’s recommendations. Genomic DNA was extracted from blood samples using the Autogen Flex Plus system (Autogen, Holliston, MA, USA). Next, the genomic DNA was quality controlled using PicoGreen (Varioskan, Thermo Fisher Scientific, Waltham, MA, USA), optical density 1.8–2.1 (Varioskan, Thermo Fisher Scientific, Waltham, MA, USA) and DNA integrity number > 6.5 (TapeStation, Agilent, Santa Clara, CA, USA). All libraries for WGS were processed with Illumina TruSEQ PCR-Free library preparation using IDT unique dual indexes. The library preparation was performed in Pre-PCR using a Hamilton NGS STAR (Hamilton, Reno, NV, USA).

### NovaSeq sequencing preparation

Normalised libraries were pooled into groups of 24 samples per run. The pooled libraries were denatured and loaded onto the NovaSEQ 6000 (Illumina, San Diego, CA, USA). All clustering was performed on the instrument using S4 flow cells. Runs were performed using 300 cycle kits with run parameters of 151, 8, 8, 151 cycles.

### HiSeq sequencing preparation

For normalisation, samples were diluted to 2.25 nM and then mixed with EPX solutions 1–3 as per the Illumina protocol. The samples were then loaded as a single sample per lane onto a cBot instrument (Illumina, San Diego, CA, USA) facilitating clustering onto the flow cell. Once complete, the flow cell was removed and loaded onto the HiSeq instrument with a 300 cycle kit with run parameters of 151, 8, 151 (no dual indexing possible).

### Variant calling

Variant calling was performed by Genuity Science Ireland (Genuity Science, Dublin, Ireland), with further outsourcing to WuXi NextCODE (WuXi NextCODE, Cambridge, USA). A Sentieon Germline pipeline (v201808.03) [30] based on the best practice of the GATK workflow was used. The pipeline involved the following steps: (1) quality check: the quality of the raw reads was checked using FASTQC (v0.11.7) (http://www.bioinformatics.babraham.ac.uk/projects/fastqc), Picard (http://broadinstitute.github.io/picard), VerifyBamID [31] and GATK [32]. (2) Alignment: a reference genome was built using human genome (assembly GRCh38; release GCA_000001405.15). Quality controlled reads were mapped to the reference genome using BWA ‘mem’ algorithm [33]. Primary and secondary alignments were taken forward for further analysis. Coverage depth was computed using the WGSMetricsAlgo (https://www.sentieon.com). (3) Post-alignment quality control was performed using several Sentieon algorithms. Specifically, alignments from the duplicate reads were marked and removed using ‘LocusCollector’ and ‘Dedup’ algorithms. Sequence alignments of indels were then refined using the ‘Realigner’ algorithm. (4) Variant calling was performed using the ‘QualCal’, ‘Haplotyper’, and ‘GVCFtyper’ Sentieon algorithms. Base quality score recalibration was performed for individual read bases of the mapped sequence read data using ‘QualCal’ algorithm, while variants were called using ‘Haplotyper’ and ‘GVCFtyper’ algorithms.

### Genetic association analyses in the GenoFit cohort

Genetic association analyses were carried out using PLINK 2.0 [34]. A generalised linear model was applied to analyse WBLM, ALM, grip strength and plasma CAF (all *n* = 6715 apart from CAF *n* = 260) as continuous traits. Covariates included age at recruitment, gender, BMI, level of education and physical activity. Ten principal components generated by PLINK were used to adjust for population stratification. The Find Irish Ancestry Computational Hunter (FIACH) score was used to exclude participants of non-Irish ancestry. The FIACH measures the Irishness of a sample using a ‘glmet’ algorithm incorporating ~ 28,710 single nucleotide polymorphisms (SNPs) selected using the UKBB. The model was trained using the reported Irish (as Irish) and non-Irish-non-UK individuals (as non-Irish) from the UKBB and then applied to the present study’s population. The results were validated by comparing the Irishness value to the reported ancestry. Additionally, related individuals were excluded based on a kinship coefficient > 0.084 (*n* = 1730). Linkage Disequilibrium (LD): PLINK was used to perform LD clumping, using the following settings: clump-p1 = 1 × 10^−4^, clump-p2 = 1 × 10^−4^, clump r2 = 0.2 and clump-kb = 1 mb. SNPs in LD with the most associated variant within the given window were grouped as one independent association.

### PhenoScanner and GenoFit replication

PhenoScanner was used to perform an initial search to determine if any *AGRN* or *PRSS12* variants have previously been associated with sarcopenia-related phenotypes. PhenoScanner is an extensive database that provides a curation of human genotype–phenotype association results from several repositories including the UKBB, the NHGRI-EBI GWAS catalogue and the Genome-Wide Repository of Associations between SNPs and Phenotypes [35, 36]. In this study, the variant-phenotype associations obtained from PhenoScanner were based solely off UKBB data. Following the search of the PhenoScanner database, the GenoFit data were consulted to identify whether the variant-phenotype associations were replicated in the GenoFit cohort. The significance threshold for replication was *p* < 0.05.

### Expression quantitative trait loci (eQTL)

The Genotype-Tissue Expression (GTEx) portal (https://gtexportal.org/home/) [37] was used for eQTL analyses to determine whether the variants were associated with a change in expression of *AGRN* and/or *PRS12*.

### Carrier analyses in the GenoFit cohort

In the GenoFit cohort, carrier analyses were performed to determine whether carriers of the *AGRN* and/or *PRSS12* variants differed phenotypically compared to non-carriers. Specifically, Jupyter notebooks [38] and SPSS software (version 26, IBM SPSS Inc., Chicago, IL, USA) were used to assess differences between carriers (AA or GA) of rs2710873 (*AGRN*) and non-carriers (GG), and between carriers (GG or GA) of rs71608359 (*PRSS12*) and non-carriers (CC). Specifically, independent samples Student’s *t*-tests, Mann–Whitney *U*-tests and analysis of covariance (ANCOVA) were performed depending on distribution normality of each variable. The phenotypes assessed included WBLM, ALM, grip strength and plasma CAF concentrations. Phenotypes were selected based off the variant-phenotype associations identified in the PhenoScanner/GenoFit analyses, with the exception of plasma CAF concentrations, which was assessed in both carrier analyses.

### Single-variant and gene-burden analyses in the UKBB

Single-variant analysis and gene-based burden tests were performed using the gene-biobank association summary statistics (Genebass) browser (https://genebass.org). Genebass is an extensive database that provides results from gene-based association analyses of ~ 3700 phenotypes in ~ 280,000 individuals with whole exome sequence (WES) data in the UKBB. The threshold for nominal significance was *p* < 0.05.

## Results

The original GenoFit data presented in this study were obtained from a total of 6715, unrelated Irish individuals aged between 18 and 83 years (males: *n* = 2997, mean age = 42.6 years; females: *n* = 3718, mean age = 46.6 years). The main characteristics of the GenoFit cohort are presented in Table 1.

**Table 1:** GenoFit cohort characteristics

Parameter	Male (*n* = 2997)	Female (*n* = 3718)	Total (*n* = 6715)
*Sociodemographic*			
Age (years)	42.6 (13.4)	46.6 (12.9)	44.8 (13.7)
*Anthropometric*			
Height (cm)	173.1 (9.9)	169.3 (9.0)	171.0 (9.6)
Body mass (kg)	77.0 (14.8)	72.2 (13.3)	74.3 (14.2)
Body mass index (kg/m^2^)	25.7 (3.7)	25.2 (3.8)	25.3 (3.4)
*Sarcopenia phenotypes*			
Grip strength (kg)	49.1 (8.5)	30.2 (5.3)	38.7 (11.7)
Appendicular lean mass (kg)	18.4 (3.9)	28.9 (2.6)	22.9 (6.0)
Whole body lean mass (kg)	60.8 (7.2)	42.2 (5.2)	50.5 (11.1)
Plasma C-terminal agrin fragment (ng/ml)^a^	2.6 (0.5)	2.7 (0.6)	2.7 (0.6)
*Lifestyle factors*			
Physical activity^b^	4.3 (2.0)	4.0 (2.0)	4.2 (2.0)
Education, *n* (%)			
No formal education	3 (0.1)	1 (< 0.0)	4 (0.1)
Primary education	22 (0.7)	21 (0.6)	43 (0.6)
Lower secondary	132 (4.4)	107 (2.9)	239 (3.6)
Higher secondary	388 (12.9)	481 (12.9)	869 (12.9)
Third-level degree	1611 (53.8)	2079 (55.9)	3690 (55.0)
Postgraduate degree	841 (28.1)	1029 (27.7)	1870 (27.8)

Data presented as means ± standard deviation unless stated otherwise; ^a^data from 260 subjects (female, *n* = 133; male, *n* = 127); ^b^days per week performing ≥ 30 min moderate intensity exercise

### *AGRN*/*PRSS12* variants and sarcopenia phenotypes in UKBB and GenoFit cohorts

According to PhenoScanner, one variant located in intron 7 of *AGRN* (rs2710873) has been previously implicated with whole body fat-free mass (WBFFM) (*β* = 0.009, *p* = 8.9 × 10^−6^) in the UKBB, while another variant located in intron 11 of *PRSS12* (rs71608359) has been associated with right hand grip strength (*β* = 0.015, *p* = 8.4 × 10^−6^), also in the UKBB (Table 2). Interestingly, we found rs2710873 and rs71608359 to be associated with broadly similar phenotypes in the GenoFit cohort. Specifically, we found rs2710873 to be nominally associated with WBLM and ALM (*β* = 0.026, *p* = 0.019 and *β* = 0.024, *p* = 0.019, respectively), while rs71608359 was nominally associated with grip strength (*β* =  − 0.042, *p* = 0.014) (Table 2). We also found rs2710873 (*AGRN*) to be associated with plasma CAF concentration (*β* =  − 0.545, *p* = 6.11 × 10^−5^) in secondary analyses combining WGS data with plasma CAF data from 260 participants of the GenoFit study. No significant association was observed between rs71608359 (*PRSS12*) and plasma CAF levels (*β* =  − 0.234, *p* = 0.254).

**Table 2:** *AGRN*/*PRSS12* variants associated with sarcopenia phenotypes in UKBB and GenoFit cohorts

rsID	Chr:position	Ref/Alt	AF	Beta	*p* value	Phenotype	Cohort	*N*
*AGRN*								
rs2710873	1:1,042,813	G/A	–	0.009	8.88 × 10^−6^	WBFFM	UKBB	331,291
rs2710873	1:1,042,813	G/A	0.179	0.026	0.019	WBLM	GenoFit	6715
rs2710873	1:1,042,813	G/A	0.179	0.024	0.019	ALM	GenoFit	6715
rs2710873	1:1,042,813	G/A	0.129	− 0.545	6.11 × 10^−5^	Plasma CAF	GenoFit	260
*PRSS12*								
rs71608359	4:118,283,390	C/G	–	0.015	8.35 × 10^−6^	Grip strength (right)	UKBB	335,842
rs71608359	4:118,283,390	C/G	0.089	− 0.042	0.014	Grip strength	GenoFit	6715
rs71608359	4:118,283,390	C/G	0.061	− 0.234	0.254	Plasma CAF	GenoFit	260

*Chr*, chromosome; *Ref/Alt*, reference allele/alternative allele; *AF*, allele frequency (unknown AF for UKBB data); *WBFFM*, whole body fat-free mass; *WBLM*, whole body lean mass; *ALM*, appendicular lean mass; *CAF*, C-terminal agrin fragment; *UKBB*, UK Biobank

#### eQTL analysis

According to the GTEx database, rs2710873 (A allele) is associated with decreased expression of *AGRN* in 10 tissues, including skeletal muscle [normalised effect size (NES) =  − 0.17, *p* = 9.6 × 10^−6^] and nerve tissue (NES =  − 0.28, 6.4 × 10^−9^) (full list in Table 3). rs71608359 (G allele) is associated with decreased *PRSS12* expression in three tissues, including thyroid (NES =  − 0.50, *p* = 5.2 × 10^−7^) and sun-exposed skin (NES =  − 0.35, *p* = 3.2 × 10^−10^) (full list in Table 3).

**Table 3:** Expression quantitative trait loci (eQTL) data for *AGRN* and *PRSS12*

rsID	eQTL (gene symbol)	Tissue	NES	*p* value
rs2710873 (A allele)	*AGRN*	Nerve—tibial	− 0.28	6.4 × 10^−9^
		Adipose—visceral	− 0.19	5.1 × 10^−8^
		Adipose—subcutaneous	− 0.19	1.5 × 10^−7^
		Artery—tibial	− 0.21	1.3 × 10^−6^
		Heart—left ventricle	− 0.19	1.5 × 10^−6^
		Artery—aorta	− 0.26	6.8 × 10^−6^
		Muscle—skeletal	− 0.17	9.6 × 10^−6^
		Oesophagus—muscularis	− 0.18	1.9 × 10^−5^
		Cells—cultured fibroblasts	− 0.17	1.5 × 10^−4^
		Thyroid	− 0.14	2.0 × 10^−4^
rs71608359 (G allele)	*PRSS12*	Skin—sun exposed	− 0.35	3.2 × 10^−10^
		Thyroid	− 0.50	5.2 × 10^−7^
		Skin—not sun exposed	− 0.24	2.3 × 10^−4^

*NES*, normalised effect size

#### Carrier analyses in the GenoFit cohort

**Fig. 1:**
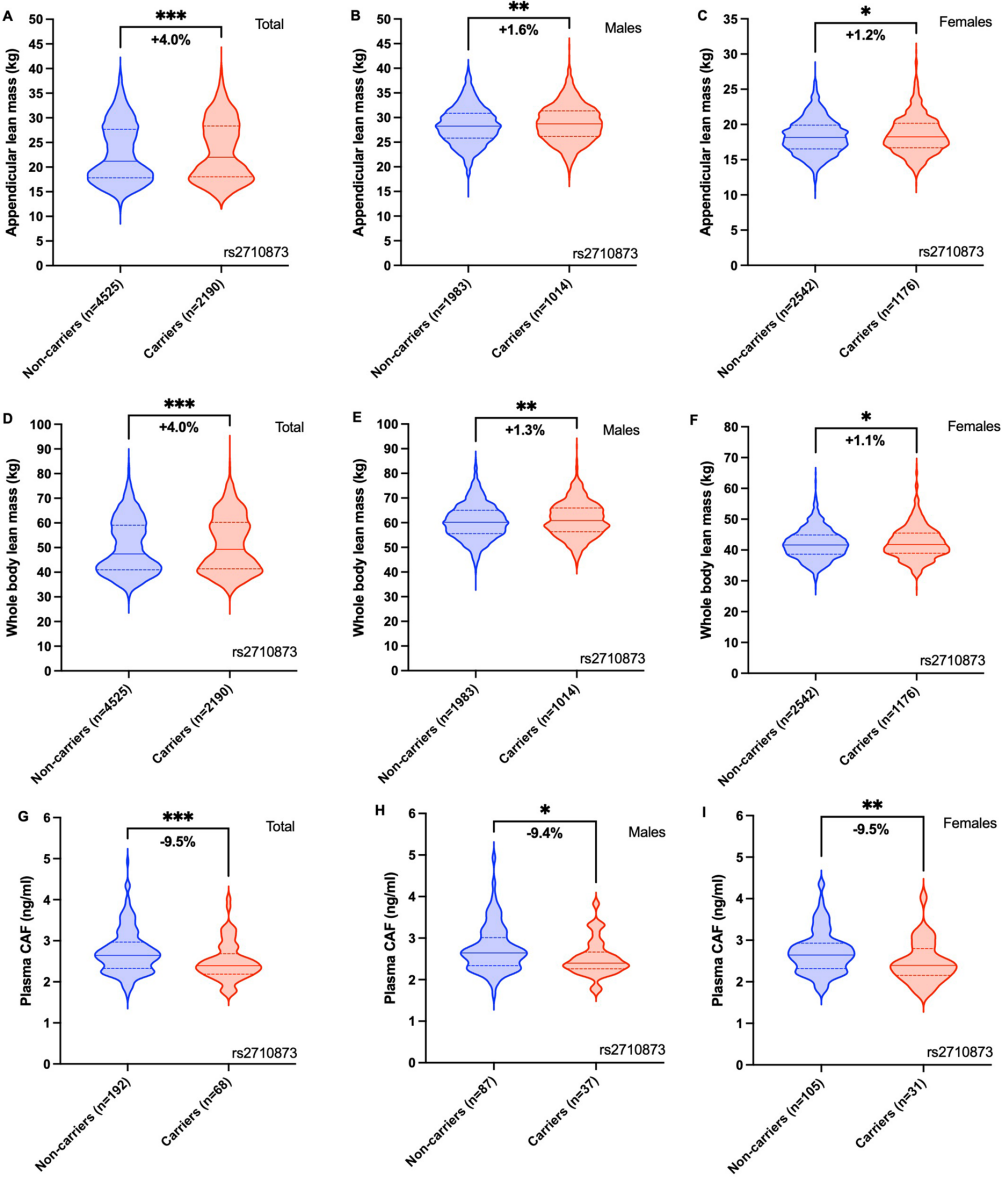
Association between carrier status of rs2710873 (*AGRN*) and; appendicular lean mass (**A** total, **B** males, **C** females), whole body lean mass (**D** total, **E** males, **F** females) and plasma C-terminal agrin fragment (CAF) (**G** total, **H** males, **I** females) in the GenoFit cohort (*p < 0.05, **p < 0.01, ***p < 0.001)

**Fig. 2:**
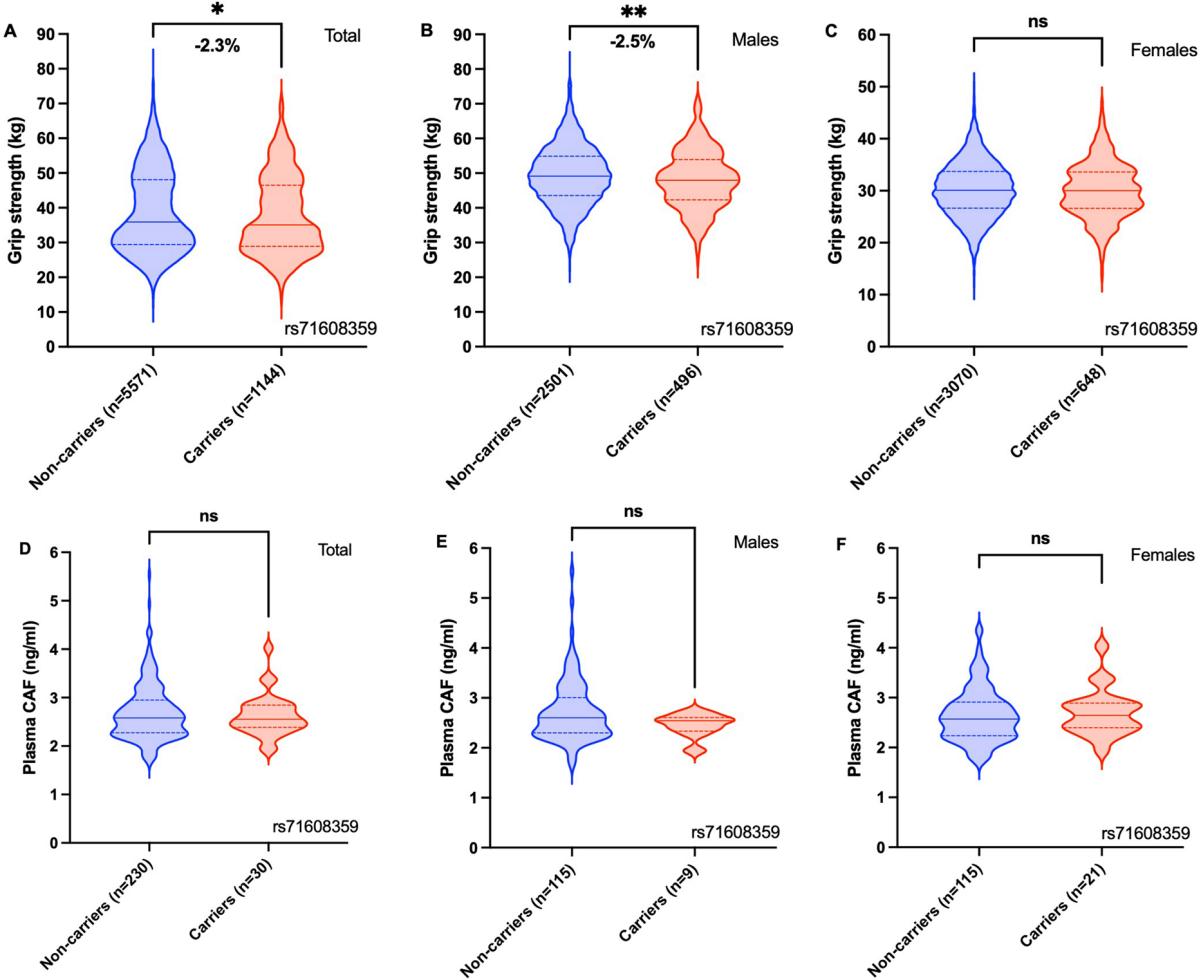
Association between carrier status of rs71608359 (*PRSS12*) and; grip strength (**A** total, **B** males, **C** females) and plasma C-terminal agrin fragment (CAF) (**D** total, **E** males, **F** females) in the GenoFit cohort (*p < 0.05, **p < 0.01)

Overall, carriers of ≥ one copy of the variant allele at rs2710873 (*AGRN*) had significantly higher WBLM and ALM (49.29 kg vs 47.41 kg, *p* < 0.001 and 22.00 kg vs 21.15 kg, *p* < 0.001, respectively), and significantly lower levels of plasma CAF (2.40 ng/ml vs 2.65 ng/ml, *p* < 0.001), compared to non-carriers (Fig. 1). Overall, carriers of ≥ one copy of the variant allele at rs71608359 (*PRSS12*) had significantly lower hand grip strength (35.08 kg vs 35.90 kg, *p* = 0.034) than non-carriers, while no significant difference in plasma CAF concentrations was observed (Fig. 2). Following stratification by sex, male and female carriers of ≥ one copy of the variant allele at rs2710873 had significantly higher WBLM (61.31 kg vs 60.54 kg, *p* = 0.006 and 42.43 kg vs 41.99 kg, *p* = 0.013, respectively) and ALM (28.73 kg vs 28.27 kg, *p* = 0.005 and 18.53 kg vs 18.31 kg, *p* = 0.014, respectively) and significantly lower plasma CAF levels (2.40 ng/ml vs 2.65 ng/ml, *p* = 0.016 and 2.39 ng/ml vs 2.64 ng/ml, *p* = 0.010, respectively), compared to non-carriers (Fig. 1). Similarly, when controlling for sex through ANCOVA, carriers of ≥ one copy of the variant allele at rs2710873 had significantly higher WBLM (50.86 kg vs 50.27 kg, *p* < 0.001) and ALM (23.14 kg vs 22.82 kg, *p* < 0.001) and significantly lower plasma CAF levels (2.49 ng/ml vs 2.73 ng/ml, *p* = 0.001), compared to non-carriers (Supplementary Table 1). Following stratification, only male carriers of ≥ one copy of the variant allele at rs71608359 had significantly lower hand grip strength (48.12 kg vs 49.35 kg, *p* = 0.003), compared to non-carriers (Fig. 2), however, adjustment for sex for through ANCOVA revealed carriers to have significantly lower hand grip strength compared to non-carriers (38.15 kg vs 38.77 kg, *p* = 0.006) (Supplementary Table 1).

#### Single-variant and gene-burden associations with sarcopenia phenotypes in the UKBB

*PRSS12 and AGRN* are also associated with grip strength and WBFFM in single-variant analyses in the UKBB. For *PRSS12*, one missense variant, rs755244092, is associated with right and left hand grip strength (*p* = 2.95 × 10^−9^ and *p* = 2.34 × 10^−8^, respectively), another missense variant, 4:118,282,033, is associated with right hand grip strength (*p* = 2.27 × 10^−9^), and a further missense variant, 4:118,313,347, is associated with WBFFM (*p* = 2.33 × 10^−5^) (Table 4). For *AGRN*, one missense variant, rs372918766, is associated with right and left hand grip strength (*p* = 4.34 × 10^−5^ and *p* = 1.03 × 10^−6^, respectively), another missense variant, rs199563268, is associated with right hand grip strength (*p* = 2.19 × 10^−5^), and a further missense variant, rs1644996826, is associated with WBFFM (*p* = 8.98 × 10^−10^) (Table 4).

**Table 4:** Single-variant analysis of *AGRN*/*PRSS12* and sarcopenia phenotypes in the UKBB

Variant ID	Consequence	Ref/Alt	AF	Beta	*p* value	Phenotype
*PRSS12*						
4:118,282,033	Missense	G/A	< 0.0001	− 4.06	2.27 × 10^−9^	Grip strength (right)
rs755244092	Missense	G/A	< 0.0001	− 4.03	2.95 × 10^−9^	Grip strength (right)
rs755244092	Missense	G/A	< 0.0001	− 3.79	2.34 × 10^−8^	Grip strength (left)
4:118,313,347	Missense	A/G	< 0.0001	− 2.52	2.33 × 10^−5^	WBFFM
*AGRN*						
rs1644996826	Missense	G/A	< 0.0001	3.65	8.98 × 10^−10^	WBFFM
rs199563268	Missense	G/A	< 0.0001	− 1.02	2.19 × 10^−5^	Grip strength (right)
rs372918766	Missense	G/C	< 0.0001	− 2.78	4.34 × 10^−5^	Grip strength (right)
rs372918766	Missense	G/C	< 0.0001	− 3.32	1.03 × 10^−6^	Grip strength (left)

*Ref/Alt*, reference allele/alternative allele; *AF*, allele frequency; *WBFFM*, whole body fat-free mass

*PRSS12* is also associated with right and left hand grip strength in gene-based burden tests (SKATO and Burden) for pLoF and synonymous variants in the UKBB (Table 5). Specifically, *PRSS12* is associated with right hand grip strength in SKATO and Burden tests of pLoF variants (both *p* < 0.001) and synonymous variants (*p* = 0.009 and *p* = 0.006, respectively). *PRSS12* is also associated with left hand grip strength in SKATO and Burden tests of synonymous variants (*p* = 0.003 and *p* = 0.002, respectively). No significant gene-burden tests were observed between *PRSS12* and muscle mass phenotypes, nor were there any between *AGRN* and grip strength or muscle mass phenotypes.

**Table 5:** Gene-burden results for *PRSS12* and grip strength in the UKBB

Variant type	Total variants	SKATO *p* value	SKAT *p* value	Burden *p* value
*Right hand grip strength*				
pLoF	97	** <** 0.001	0.051	** <** 0.001
Missense	520	0.129	0.074	0.277
Synonymous	191	0.009	0.255	0.006
*Left hand grip strength*				
pLoF	97	0.141	0.125	0.088
Missense	519	0.556	0.359	0.773
Synonymous	191	0.003	0.094	0.002

## Discussion

Despite strong physiological evidence supporting the importance of the NMJ in maintaining skeletal muscle health during ageing, genetic studies relating to sarcopenia have identified few genes involved in NMJ function. Furthermore, although agrin and neurotrypsin are considered to be fundamental for NMJ function, few studies have explored the relevance of *AGRN* and *PRSS12* to sarcopenia. In light of this, we aimed to determine whether *AGRN* and/or *PRSS12* were associated with sarcopenia phenotypes including muscle mass, strength and plasma CAF levels.

Collectively, our findings support the relevance of agrin and neurotrypsin to sarcopenia, and provide novel evidence of the pertinence of *AGRN* and *PRSS12* to sarcopenia phenotypes. We identified two variants, rs2710873 (*AGRN*) and rs71608359 (*PRSS12*), that were associated with sarcopenia phenotypes in the UKBB and GenoFit cohorts (Table 2). Interestingly, rs2710873 and rs71608359 are eQTLs for *AGRN* and *PRSS12*, respectively, in ≥ three tissues, highlighting their functional impact on gene expression (Table 3). Findings from carrier analyses further support the relevance of these particular variants to sarcopenia phenotypes. For example, in the GenoFit cohort, carriers of rs2710873 had significantly higher WBLM and ALM, and significantly lower plasma CAF concentrations, compared to non-carriers (Fig. 1). This is a particularly important finding as it supports existing physiological data that illustrate an inverse relationship between circulating CAF concentrations and skeletal muscle health [13, 25, 39]. Interestingly, while carriers of the rs71608359 variant had significantly lower grip strength than non-carriers (only for the male subjects), no significant difference in plasma CAF levels was observed (Fig. 2). This is reflected by the GenoFit data, whereby strong associations were observed between rs2710873 (*AGRN*) and plasma CAF (*p* = 6.11 × 10^−5^), but no such association was found between rs71608359 (*PRSS12*) and plasma CAF (*p* = 0.254) (Table 2). Thus, it seems the underpinning mechanism driving the association between this particular *PRSS12* variant and grip strength, may be separate to the pathway of neurotrypsin-dependent agrin cleavage. It is noteworthy, however, that the sample of circulating CAF data collected in the present study was small (*n* = 260), and so, larger studies are needed to contextualise our findings.

The single-variant and gene-burden data obtained from UKBB WES data further support the potential relevance of *AGRN* and *PRSS12* to sarcopenia. Indeed, there are several missense variants located in the coding region of *AGRN* and *PRSS12* that associate with muscle strength and mass phenotypes (Table 4), and gene-burden data that indicate the combined effect of rare *PRSS12* variants associating with muscle strength (Table 5). Such WES data are an important aspect to elucidating gene-phenotype associations, as it is particularly useful for examining the effect of rare-coding variants on highly complex phenotypes, such as muscle mass and strength. Furthermore, increasing evidence suggests that there is an appreciable contribution of rare variants to heritability of human traits and diseases [40, 41]. Therefore, the single-variant and gene-burden results complement the WGS data, illustrating that both common intronic and rare exonic *AGRN*/*PRSS12* variants associate with phenotypes relevant to sarcopenia.

Interestingly, the single-variant analysis revealed two variants, 4:118,282,033 (*PRSS12*) and rs199563268 (*AGRN*) associated with right hand grip strength, but not left hand. Unfortunately, the current paucity of data surrounding potential differences in the genetic architecture of left hand vs right hand grip strength impedes our interpretation of these findings. This dearth is somewhat due to existing studies considering only the mean grip strength of both hands [9] or maximum grip strength from either hand [42, 43], eliminating the potential to elucidate genetic differences between hands. Interestingly however, a recent study based upon UKBB data identified 160 SNPs for right hand grip strength and 136 SNPs for left hand grip strength [44], demonstrating hand-specific differences in the genetic basis of grip strength. Considering approximately 90% of the global population are right-handed [45], it is plausible that this phenomenon is somewhat due to dominant hand grip strength having a greater genetic architecture than the non-dominant hand. Nevertheless, although handedness is a notably heritable trait [46], whether the genetic underpinnings of dominant vs non-dominant hand grip strength differ remains to be seen. In this regard, further studies considering data from both hands independently are needed to illuminate the potential mediating effect of handedness on the genetic foundations of grip strength.

Notwithstanding the UKBB data, there are limited published data concerning the potential associations between *AGRN*/*PRSS12* variants and sarcopenia phenotypes. There is, however, evidence of interactions between these genes and other potentially relevant phenotypes. Firstly, several *AGRN* variants have been associated with neurological and neuromuscular disorders [47–49], including congenital myasthenic syndromes (CMS), a rare group of diseases caused by a severe impairment of synaptic transmission at the NMJ [50]. Considering CMS presents as a fatiguing weakness of ocular and cranial muscles that later generalises to weakness and wasting of limb muscles, it seems plausible that *AGRN* may also relate to generalised skeletal muscle deterioration as part of the sarcopenic phenotype. This is supported by studies showing that the expression of *AGRN* is significantly increased in several muscles of old vs young/adult mice [51] and in human vastus lateralis muscle following 10 days of bed rest [52]. Importantly, in both studies, the increased expression of *AGRN* was accompanied by phenotypic changes relevant to sarcopenia, such as NMJ fragmentation, reduction in muscle cross-sectional area and contractile potential, and denervation. Secondly, despite being most extensively implicated with intellectual ability, there is also evidence supporting the role of *PRSS12* in skeletal muscle regulation during ageing. For example, studies have observed the onset of sarcopenia in old mice to be accompanied by a significant increase in *PRSS12* expression [53] while, as mentioned previously, another study reported over-expression of *PRSS12* to induce NMJ fragmentation and precocious sarcopenia [26]. Changes in *PRSS12* expression have also been associated with extracellular matrix remodelling [54], a potential contributor to the development of sarcopenia [55]. Hence, despite there being no evidence directly linking *PRSS12* variants and sarcopenia, the change in *PRSS12* expression in several phenotypes relevant to sarcopenia is promising.

Interestingly, *AGRN* has been shown to be upregulated in mouse soleus muscle following resistance training [56], while a recent transcriptomic meta-analysis suggests that *AGRN* is also upregulated in human vastus lateralis muscle in response to resistance training and/or combined training (resistance training and aerobic training) [57]. While upon first consideration it seems counterintuitive that inactivity and training interventions would both each induce an upregulation of *AGRN*, the context in which these changes occur is fundamental. For example, while the increase of *AGRN* following inactivity is due to a negative breakdown of neuromuscular integrity, the increase following training is likely a facilitatory response to promote positive neuromuscular remodelling. Unlike *AGRN*, there are currently no data suggesting an exercise related mediation of *PRSS12* expression. Nevertheless, it is noteworthy that the effects of *AGRN* and *PRSS12* on sarcopenia phenotypes are likely to be synergistic, whereby changes in *PRSS12* expression may stimulate changes in *AGRN* expression. Indeed, the increased *AGRN* expression observed in degrading muscle appears to be a compensatory response to increased neurotrypsin-dependent cleavage of agrin at the NMJ. This hypothesis is supported by data showing that an increase in *AGRN* expression is accompanied by an increase in circulating CAF [52]. While several factors may contribute to the change in *AGRN* expression, including a potentially compensatory response to changes in the degree of agrin cleavage at the NMJ, it seems logical for changes in *PRSS12* expression to be a principal contributor. Considering that no significant association was observed between rs71608359 (*PRSS12*) and CAF concentrations in the present study, it is clear that more research is needed to further elucidate the extent of the interplay between these genes.

There are several strengths and limitations to our study that should be discussed. The main strengths of this study include the use of WGS and the incorporation of an extensively phenotyped study sample spanning the entire adult lifespan. Furthermore, participant data were obtained at only two sites, by identically trained study personnel, further enhancing the quality of data. The first and main limitation of our study is the relatively small sample size of the GenoFit cohort, particularly in relation to samples with plasma CAF data. While promising associations have been uncovered in this study, much larger samples are needed to confirm our findings. In this regard, future studies should seek to confirm the effect direction of the variants identified in this study. For example, while the effect direction was positive for rs2710873 in the UKBB and GenoFit cohort, for rs71608359 it was positive in the UKBB and negative in the GenoFit cohort (Table 2). Although nuances in methodology may contribute to these differences, ameliorating the current paucity of data in this area is critical to providing further insight into the physiological effect of the identified variants. Secondly, plasma CAF concentrations were only determined for 260 participants, and so results relating to this marker should be interpreted accordingly. Thirdly, while in the present study we selected *AGRN* and *PRSS12* as modulators of NMJ health, many other genes are known to effect NMJ function and should be considered in future research. Finally, the study was conducted only in those from Irish descent, and so, the transferability of findings to other populations is unknown.

In conclusion, despite the current dearth of evidence, *AGRN* and *PRSS12* appear to be promising candidate genes for sarcopenia. Our findings support their relevance to sarcopenia phenotypes and support existing physiological data that indicate that the NMJ is a central mediator of skeletal muscle health. We hope our findings encourage future endeavours to further explore the potential role of *AGRN* and *PRSS12* in sarcopenia pathogenesis. In this regard, there is a strong need for larger studies with well-characterised and homogenously analysed cohorts to confirm our findings.

## Supplementary Information

Below is the link to the electronic supplementary material.

Supplementary file1 (DOCX 14 KB)

## Data Availability

Data may be made available upon reasonable request to the corresponding author.

## References

[1] Rosenberg IH (1997). Sarcopenia: origins and clinical relevance. J Nutr.

[2] Cruz-Jentoft AJ, Bahat G, Bauer J, Boirie Y, Bruyère O, Cederholm T (2019). Sarcopenia: revised European consensus on definition and diagnosis. Age Ageing.

[3] Landi F, Liperoti R, Russo A, Giovannini S, Tosato M, Capoluongo E (2012). Sarcopenia as a risk factor for falls in elderly individuals: results from the ilSIRENTE study. Clin Nutr.

[4] Janssen I, Heymsfield SB, Ross R (2002). Low relative skeletal muscle mass (sarcopenia) in older persons is associated with functional impairment and physical disability. J Am Geriatr Soc.

[5] Dos Santos L, Cyrino ES, Antunes M, Santos DA, Sardinha LB (2017). Sarcopenia and physical independence in older adults: the independent and synergic role of muscle mass and muscle function. J Cachexia Sarcopenia Muscle.

[6] Shafiee G, Keshtkar A, Soltani A, Ahadi Z, Larijani B, Heshmat R (2017). Prevalence of sarcopenia in the world: a systematic review and meta-analysis of general population studies. J Diabetes Metab Disord.

[7] Abney M, McPeek MS, Ober C (2001). Broad and narrow heritabilities of quantitative traits in a founder population. Am J Hum Genet.

[8] Zempo H, Miyamoto-Mikami E, Kikuchi N, Fuku N, Miyachi M, Murakami H (2017). Heritability estimates of muscle strength-related phenotypes: a systematic review and meta-analysis. Scand J Med Sci Sports.

[9] Tikkanen E, Gustafsson S, Amar D, Shcherbina A, Waggott D, Ashley EA (2018). Biological insights into muscular strength: genetic findings in the UK Biobank. Sci Rep.

[10] Zillikens MC, Demissie S, Hsu YH, Yerges-Armstrong LM, Chou WC, Stolk L (2017). Large meta-analysis of genome-wide association studies identifies five loci for lean body mass. Nat Commun.

[11] Jones G, Trajanoska K, Santanasto AJ, Stringa N, Kuo CL, Atkins JL (2021). Genome-wide meta-analysis of muscle weakness identifies 15 susceptibility loci in older men and women. Nat Commun.

[12] Pei YF, Liu YZ, Yang XL, Zhang H, Feng GJ, Wei XT (2020). The genetic architecture of appendicular lean mass characterized by association analysis in the UK Biobank study. Commun Biol.

[13] Pratt J, De Vito G, Narici M, Segurado R, Pessanha L, Dolan J (2021). Plasma C-terminal agrin fragment as an early biomarker for sarcopenia: results from the GenoFit study. J Gerontol A Biol Sci Med Sci.

[14] Tintignac LA, Brenner HR, Ruegg MA (2015). Mechanisms regulating neuromuscular junction development and function and causes of muscle wasting. Physiol Rev.

[15] Pratt J, De Vito G, Narici M, Boreham C (2021). Neuromuscular junction aging: a role for biomarkers and exercise. J Gerontol A Biol Sci Med Sci.

[16] Wilson D, Jackson T, Sapey E, Lord JM (2017). Frailty and sarcopenia: the potential role of an aged immune system. Ageing Res Rev.

[17] Pratt J, Boreham C, Ennis S, Ryan AW, De Vito G (2019). Genetic associations with aging muscle: a systematic review. Cells.

[18] Morley JE, Argiles JM, Evans WJ, Bhasin S, Cella D, Deutz NE (2010). Nutritional recommendations for the management of sarcopenia. J Am Med Dir Assoc.

[19] Pratt J, De Vito G, Segurado R, Pessanha L, Dolan J, Narici M (2022). Plasma neurofilament light levels associate with muscle mass and strength in middle-aged and older adults: findings from GenoFit. J Cachexia Sarcopenia Muscle.

[20] Lepore E, Casola I, Dobrowolny G, Musarò A (2019). Neuromuscular junction as an entity of nerve-muscle communication. Cells.

[21] Hepple RT, Rice CL (2016). Innervation and neuromuscular control in ageing skeletal muscle. J Physiol.

[22] Piasecki M, Ireland A, Piasecki J, Stashuk DW, Swiecicka A, Rutter MK (2018). Failure to expand the motor unit size to compensate for declining motor unit numbers distinguishes sarcopenic from non-sarcopenic older men. J Physiol.

[23] Stephan A, Mateos JM, Kozlov SV, Cinelli P, Kistler AD, Hettwer S (2008). Neurotrypsin cleaves agrin locally at the synapse. Faseb J.

[24] Kim N, Stiegler AL, Cameron TO, Hallock PT, Gomez AM, Huang JH (2008). Lrp4 is a receptor for agrin and forms a complex with MuSK. Cell.

[25] Landi F, Calvani R, Lorenzi M, Martone AM, Tosato M, Drey M (2016). Serum levels of C-terminal agrin fragment (CAF) are associated with sarcopenia in older multimorbid community-dwellers: results from the ilSIRENTE study. Exp Gerontol.

[26] Butikofer L, Zurlinden A, Bolliger MF, Kunz B, Sonderegger P (2011). Destabilization of the neuromuscular junction by proteolytic cleavage of agrin results in precocious sarcopenia. Faseb j.

[27] Bolliger MF, Zurlinden A, Lüscher D, Bütikofer L, Shakhova O, Francolini M (2010). Specific proteolytic cleavage of agrin regulates maturation of the neuromuscular junction. J Cell Sci.

[28] Hettwer S, Lin S, Kucsera S, Haubitz M, Oliveri F, Fariello RG (2014). Injection of a soluble fragment of neural agrin (NT-1654) considerably improves the muscle pathology caused by the disassembly of the neuromuscular junction. PLoS ONE.

[29] Pratt J, De Vito G, Narici M, Segurado R, Dolan J, Conroy J (2021). Grip strength performance from 9431 participants of the GenoFit study: normative data and associated factors. Gerosci.

[30] Freed D, Aldana R, Weber JA, Edwards JS (2017). The Sentieon Genomics Tools - a fast and accurate solution to variant calling from next-generation sequence data. bioRxiv.

[31] Jun G, Flickinger M, Hetrick KN, Romm JM, Doheny KF, Abecasis GR (2012). Detecting and estimating contamination of human DNA samples in sequencing and array-based genotype data. Am J Hum Genet.

[32] McKenna A, Hanna M, Banks E, Sivachenko A, Cibulskis K, Kernytsky A (2010). The Genome Analysis Toolkit: a MapReduce framework for analyzing next-generation DNA sequencing data. Genome Res.

[33] Li H. Aligning sequence reads, clone sequences and assembly contigs with BWA-MEM. arXiv preprint. 2013;1303.3997v1

[34] Purcell S, Neale B, Todd-Brown K, Thomas L, Ferreira MA, Bender D (2007). PLINK: a tool set for whole-genome association and population-based linkage analyses. Am J Hum Genet.

[35] Staley JR, Blackshaw J, Kamat MA, Ellis S, Surendran P, Sun BB (2016). PhenoScanner: a database of human genotype-phenotype associations. Bioinformatics.

[36] Kamat MA, Blackshaw JA, Young R, Surendran P, Burgess S, Danesh J (2019). PhenoScanner V2: an expanded tool for searching human genotype-phenotype associations. Bioinformatics.

[37] GTExConsortium (2015). Human genomics. The Genotype-Tissue Expression (GTEx) pilot analysis: multitissue gene regulation in humans. Science.

[38] Kluyver T, Ragan-Kelley B, Pérez F, Granger B, Bussonnier M, Frederic J, et al. Jupyter Notebooks – a publishing format for reproducible computational workflows. Positioning and power in academic publishing: players, agents and agendas. 2016;87–90

[39] Sarto F, Stashuk DW, Franchi MV, Monti E, Zampieri S, Valli G (2022). Effects of short-term unloading and active recovery on human motor unit properties, neuromuscular junction transmission and transcriptomic profile. J Physiol.

[40] Mancuso N, Rohland N, Rand KA, Tandon A, Allen A, Quinque D (2016). The contribution of rare variation to prostate cancer heritability. Nat Genet.

[41] Wang Q, Dhindsa RS, Carss K, Harper AR, Nag A, Tachmazidou I (2021). Rare variant contribution to human disease in 281,104 UK Biobank exomes. Nature.

[42] Willems SM, Wright DJ, Day FR, Trajanoska K, Joshi PK, Morris JA (2017). Large-scale GWAS identifies multiple loci for hand grip strength providing biological insights into muscular fitness. Nat Commun.

[43] Sarnowski C, Chen H, Biggs ML, Wassertheil-Smoller S, Bressler J, Irvin MR (2021). Identification of novel and rare variants associated with handgrip strength using whole genome sequence data from the NHLBI Trans-Omics in Precision Medicine (TOPMed) Program. PLoS ONE.

[44] Zhuo C, Zhao J, Wang Q, Lin Z, Cai H, Pan H (2022). Assessment of causal associations between handgrip strength and cardiovascular diseases: a two sample mendelian randomization study. Front Cardiovasc Med.

[45] Papadatou-Pastou M, Ntolka E, Schmitz J, Martin M, Munafò MR, Ocklenburg S (2020). Human handedness: a meta-analysis. Psychol Bull.

[46] Medland SE, Duffy DL, Wright MJ, Geffen GM, Hay DA, Levy F (2009). Genetic influences on handedness: data from 25,732 Australian and Dutch twin families. Neuropsychol.

[47] Wightman DP, Jansen IE, Savage JE, Shadrin AA, Bahrami S, Holland D (2021). A genome-wide association study with 1,126,563 individuals identifies new risk loci for Alzheimer’s disease. Nat Genet.

[48] Nicole S, Chaouch A, Torbergsen T, Bauché S, de Bruyckere E, Fontenille MJ (2014). Agrin mutations lead to a congenital myasthenic syndrome with distal muscle weakness and atrophy. Brain.

[49] Topaloudi A, Zagoriti Z, Flint AC, Martinez MB, Yang Z, Tsetsos F (2021). Myasthenia gravis genome-wide association study implicates AGRN as a risk locus. J Med Genet.

[50] Engel AG, Shen XM, Selcen D, Sine SM (2015). Congenital myasthenic syndromes: pathogenesis, diagnosis, and treatment. Lancet Neurol.

[51] Blasco A, Gras S, Mòdol-Caballero G, Tarabal O, Casanovas A, Piedrafita L (2020). Motoneuron deafferentation and gliosis occur in association with neuromuscular regressive changes during ageing in mice. J Cachexia Sarcopenia Muscle.

[52] Monti E, Reggiani C, Franchi MV, Toniolo L, Sandri M, Armani A (2021). Neuromuscular junction instability and altered intracellular calcium handling as early determinants of force loss during unloading in humans. J Physiol.

[53] Barns M, Gondro C, Tellam RL, Radley-Crabb HG, Grounds MD, Shavlakadze T (2014). Molecular analyses provide insight into mechanisms underlying sarcopenia and myofibre denervation in old skeletal muscles of mice. Int J Biochem Cell Biol.

[54] Chapman MA, Mukund K, Subramaniam S, Brenner D, Lieber RL (2017). Three distinct cell populations express extracellular matrix proteins and increase in number during skeletal muscle fibrosis. Am J Physiol Cell Physiol.

[55] Melouane A, Yoshioka M, St-Amand J (2020). Extracellular matrix/mitochondria pathway: a novel potential target for sarcopenia. Mitochondrion.

[56] Dungan CM, Brightwell CR, Wen Y, Zdunek CJ, Latham CM, Thomas NT (2022). Muscle-specific cellular and molecular adaptations to late-life voluntary concurrent exercise. Function (Oxf).

[57] Pillon NJ, Gabriel BM, Dollet L, Smith JAB, Sardón Puig L, Botella J (2020). Transcriptomic profiling of skeletal muscle adaptations to exercise and inactivity. Nat Commun.

