# Supplementary material for: Genes encoding agrin (AGRN) and neurotrypsin (PRSS12) are associated with muscle mass, strength and plasma C-terminal agrin fragment concentration
Source: GeroScience. 2023 Jan 7;45(3):1289–302. doi: 10.1007/s11357-022-00721-1 (PMC10400504; doi:10.1007/s11357-022-00721-1)
Supplement: Supplementary file 1 — Supplementary file1 (DOCX 14 KB) [file 11357_2022_721_MOESM1_ESM.docx]

| Supplementary Table 1. Association between carrier status of *PRSS12* / *AGRN* variants and sarcopenia phenotypes adjusted for sex | | | |
| --- | --- | --- | --- |
| Phenotype | Carriers | Non-carriers | p-value |
| *rs71608359 (PRSS12)* | | | |
| C-terminal agrin fragment (ng/ml) | 2.63 (0.10) | 2.68 (0.04) | 0.644 |
| Grip strength (kg) | 38.15 (0.20) | 38.77 (0.09) | 0.006 |
| *rs2710873 (AGRN)* | | | |
| Appendicular lean mass (kg) | 23.14 (0.07) | 22.82 (0.05) | <0.001 |
| Whole body lean mass (kg) | 50.86 (0.13) | 50.27 (0.09) | <0.001 |
| C-terminal agrin fragment (ng/ml) | 2.49 (0.06) | 2.73 (0.04) | 0.001 |
| Data presented as mean (standard error of the mean); total group rs71608359 carriers = 1144 vs non-carriers = 5571, and rs2710873 carriers = 2190 vs non-carriers = 4525; C-terminal agrin fragment subgroup rs71608359 carriers = 30 vs non-carriers = 230, and rs2710873 carriers = 68 vs non-carriers = 192 | | | |
